# Role of LIMK1-cofilin-actin axis in dendritic spine dynamics in Alzheimer’s disease

**DOI:** 10.1038/s41419-025-07741-7

**Published:** 2025-06-03

**Authors:** Fabiola Paciello, Martina Battistoni, Sara Martini, Chiara Simone, Francesco Pastore, Raimondo Sollazzo, Claudio Grassi, Cristian Ripoli

**Affiliations:** 1 Department of Neuroscience, Università Cattolica del Sacro Cuore, Rome, Italy; 2 Fondazione Policlinico Universitario A. Gemelli IRCCS, Rome, Italy

**Keywords:** Spine regulation and structure, Molecular neuroscience

## Abstract

Dysregulation of dendritic spine dynamics, a process essential for synaptic plasticity and memory, is a hallmark of Alzheimer’s disease (AD). Actin dynamics, largely regulated by the LIMK1-cofilin pathway, are central to maintaining structural and functional stability in neurons. In healthy brains, the LIMK1-cofilin-actin axis modulates actin polymerization within dendritic spines, supporting spine growth and plasticity. However, in AD, this pathway is altered, leading to both actin and synaptic dysfunction. Studies report conflicting findings, with some indicating LIMK1 hyperactivation leading to cofilin inactivation, while others observe elevated cofilin activity, suggesting divergent regulatory mechanisms depending on the disease stage or neuronal environment. The paradoxical effects of LIMK1-cofilin signaling in AD may result from a context-dependent regulation influenced by factors such as amyloid-beta (Aβ) and tau protein accumulation, which disrupt actin dynamics and promote synaptic degeneration. The presence of cofilin-actin rods and Hirano bodies in AD highlights the role of aberrant actin stabilization and its impact on neurodegenerative processes. This review synthesizes current findings on LIMK1-cofilin-actin signaling in AD, emphasizing the dual role of cofilin in stabilizing and severing actin filaments. Targeting the LIMK1-cofilin-actin axis presents a promising therapeutic approach to restore dendritic spine dynamics and mitigate cognitive decline. However, resolving inconsistencies in cofilin regulation is essential to developing effective treatments for AD.

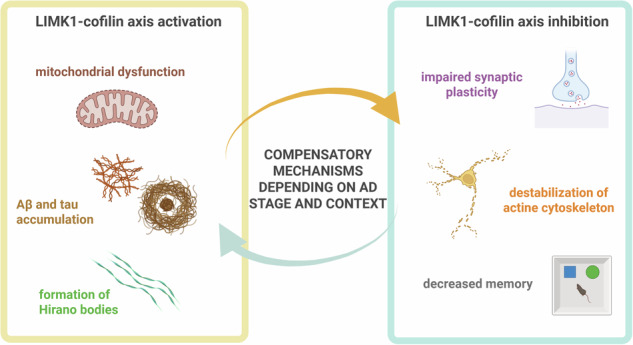

## Facts

- LIMK1 play a key role in structural plasticity phosphorylating substrates like cofilin, thereby regulating actin filament dynamics in spines.
- The balance between phosphorylated (inactive) and dephosphorylated (active) cofilin is crucial for regulating actin dynamics, synaptic plasticity, and structural changes in spines.
- Dysregulation of LIMK1-cofilin-actin axis has been implicated in AD pathology.
- Targeting cofilin regulation through LIMK1 inhibitors or SSH activators presents a promising therapeutic strategy for AD.

## Open Questions

- What are the molecular mechanisms driving the biphasic pattern of cofilin phosphorylation in AD progression?
- How does LIMK1-cofilin-actin dysregulation impact dendritic spine dynamics across different stages of AD?
- Can LIMK1 inhibitors be safely used in humans without disrupting essential physiological processes involving actin dynamics?

## Introduction

**Fig. 1:**
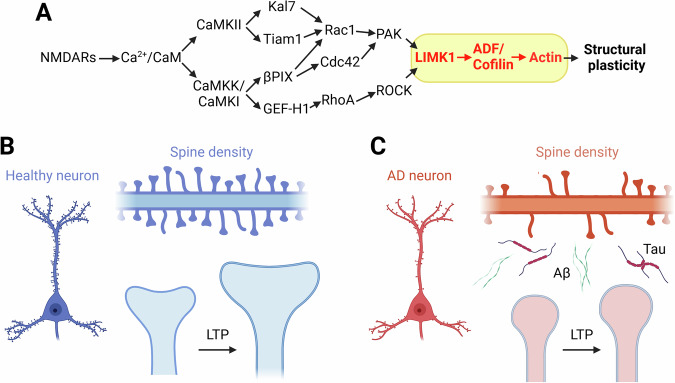
Synaptic and structural plasticity in healthy and AD neurons. **A** Pathway diagram of canonical signaling involved in structural plasticity of dendritic spines. In AD, this pathway is disrupted, leading to compromised synaptic and structural plasticity (modified from Ripoli et al. [90]). **B** Illustration of a healthy neuron and its spine density. Dendritic spine schematic representation on the right shows high spine density and synaptic growth during LTP in healthy neurons, indicating robust structural plasticity. **C** Cartoon of an AD neuron and its reduced spine density compared with A. The presence of amyloid-β (Aβ) protein and tau oligomers is depicted, which contributes to impaired structural plasticity of dendritic spines, leading to diminished synaptic connections and plasticity. Created with BioRender.com.

Among neurodegenerative disorders, Alzheimer’s disease (AD) is characterized by progressive cognitive decline and memory loss [1, 2]. Indeed, a significant pathological feature of AD is the loss of dendritic spines, especially in brain areas involved in cognitive functions, such as the hippocampus [3]. Dendritic spines are small, actin-rich protrusions on neuronal dendrites containing glutamatergic receptors that mediate excitatory synaptic transmission and plasticity [4, 5]. Dynamic modulation of actin polymerization within spines is crucial for the structural changes associated with volume enlargement and the parallel increase of α-amino-3-hydroxy-5-methyl-4-isoxazolepropionic acid receptors (AMPAR) on the post-synaptic site. Indeed, filamentous actin (F-actin) crosslinks with surface receptors and integral post-synaptic proteins, such as Drebrin and PSD95 [6], play a key role in determining spine structure. Dynamic modulation of actin polymerization in spines is closely linked to learning and memory [7]. Thus, actin-mediated structural plasticity and functional long-term potentiation (LTP), which involve activity-dependent enhancement of neuronal communication, are strictly related [8]. Dysregulation of these processes has been implicated in AD [9]; however, the exact mechanisms remain unclear. The canonical molecular pathway responsible for dendritic spine plasticity encompasses dozens of proteins (most of them summarized in Table 1) that are compartmentalized within these structures, thereby facilitating localized signal transduction [10]. Within this intricate molecular network, an actin regulatory protein plays a crucial role: the serine/threonine LIM (Lin-11/Isl-1/Mec-3) domain-containing protein kinase 1 (LIMK1), which can inactivate the actin-severing protein cofilin, preventing the cleavage of F-actin, stabilizing the actin cytoskeleton, and promoting actin polymerization and spine enlargement [11] (Fig. 1A, B). The activity of LIMK1 is regulated by Rho family GTPases, including Ras-related C3 botulinum toxin substrate 1 (Rac1), Cell division cycle 42 (Cdc42), and Ras homolog family member A (RhoA). These GTPases exert their effects through the activity of specific effectors, Rho-associated protein kinase (ROCK) and p21-activated kinase (PAK1), which phosphorylate LIMK1 at threonine 508 (T508) within its activation loop, thereby activating LIMK1 [12] (Fig. 1A). Ca^2+^influx through N-methyl-D-aspartate receptors (NMDAR) induces the activation of Rho-GTPases, which become permeable when the high-frequency activity of pre- and post-synaptic neurons coincides, subsequently triggering Ca^2+^/calmodulin-dependent protein kinase II (CaMKII) [13].

**Table 1:** Major proteins involved in spine dynamics.

Protein	Physiological role	Pathological role
Drebrin	Actin-binding protein that stabilizes F-actin [92].	Decreased levels of Drebrin in temporal and frontal cortex of AD brains [93]
TIAM1	Regulates Rac1 GDP/GTP exchange inducing Rac1 activation [94]	*Tiam1* KO mice correlate with reduced dendritic spine density and arborization and reduced excitatory synaptic transmission [95]
Rac1	Promotes LIMK1 activation *via* PAK phosphorylation [8, 11].	Increased Rac1 expression in cortex of AD brain [96]
Cdc42	Promotes LIMK1 activation *via* PAK phosphorylation [8, 11].	Increased Cdc42 expression in cortex of AD brain [96]
PAK	Phosphorylation and activation of LIMK1 at Thr-508 [8, 11].	Loss of PAK1in hippocampus of AD brain and loss of Pak3 both in hippocampus and cortex in AD brain [97]
RhoA	Promotes LIMK1 activation *via* ROCK phosphorylation [8, 11].	Hyperactivation of RhoA pathway increases β-secretase and γ-secretase activity [98] RhoA pathway overactivation promotes abnormal tau protein phosphorylation [98]
ROCK	Phosphorylates and activates LIMK1 at Thr-508 [8, 11].	Hyperactivation of ROCK pathway increases β-secretase and γ-secretase activity [98] ROCK pathway overactivation promotes abnormal tau protein phosphorylation [98]
LIMK1	ADF/Cofilin phosphorylation on Ser-3 promoting the stabilization of dendritic spines [8, 11].	LIMK1 hyperphosphorylation is associated with dystrophic neurites [17].
Cofilin	Induces actin filament stabilization when phosphorylated by LIMK1 on Ser-3 (inactive form) [8, 11].	Formation of cofilin rods (Barone et al., 2014); Aβ- and tau interaction inducing mitochondrial and synaptic dysfunctions [72, 73]. Hirano bodies [22, 24].
F-actin	Regulates spine remodeling (i.e. enlargement) due to its polymerization [8, 11].	Cofilin-actin rods [66]

Activated LIMK1 then phosphorylates cofilin at serine 3 (Ser3),the major cytoskeleton protein within dendritic spines, and the LIMK1-cofilin interaction is a critical process for the modulation of spine structure and function (Fig. 1A). This phosphorylation, leading to cofilin inactivation, is reversed by the F-actin-associated protein phosphatase Slingshot-1 (SSH1), leading to the reactivation of cofilin [14].

LIMK1-cofilin-actin axis dysregulation has been implicated in AD [15, 16]; however, experimental studies have led to inconsistent results and different conclusions. Therefore, whether the axis is inactivated or hyperactivated in AD pathology remains unclear. Notably, an abnormal increase in phosphorylated LIMK1, which enhances cofilin phosphorylation and inhibits its activity, thus potentially promoting actin polymerization, has been linked to neuronal dystrophy observed in the brain regions of patients affected by AD (Fig. 1C) [17]. Similarly, pharmacological inhibition of LIMK1 has been shown to provide dendritic spine resilience against amyloid-β (Aβ) protein, the main constituent of amyloid plaques and one of the core hallmarks of AD, together with neurofibrillary tangle accumulation, consisting of hyperphosphorylated tau protein [18]. In contrast, Aβ oligomers have been shown to promote cofilin dephosphorylation, leading to synaptic protein loss, deficits in LTP, and altered contextual memory in APP/PS1 mice, an experimental model of AD [19]. Additionally, Aβ42-induced spine loss, observed after a 10-day exposure to Aβ oligomers, can be blocked by the expression of constitutively inactive cofilin (S3D, in which Ser3 is replaced by the phosphomimetic amino acid aspartate) [20]. Kim et al. [21] found significantly reduced levels of phosphorylated cofilin in the frontal cortex of patients with AD compared to healthy controls, suggesting elevated cofilin activity [21], which appears to contradict the findings of Heredia and colleagues [17].

Of note, cofilin is one of the main components of Hirano bodies, which are intracellular rod-shaped inclusions found in both neurons and glia of the central nervous system [22]. These structures primarily consist of actin, cofilin, and other cytoskeletal proteins, such as tau, middle molecular weight neurofilament subunits, and a C-terminal fragment of β-amyloid precursor protein (APP) [23]. Hirano bodies are commonly associated with neurodegenerative diseases, especially AD, and are often located in brain regions regulating memory and cognitive function, such as the hippocampus [22, 24]. Similarly, cofilin-actin rods, such as Hirano bodies, are also found in AD brains [25, 26], indicating a possible causal or consequential dysfunction of cofilin and actin in neurons of patients with AD. These rod-like inclusions suggested that the normal dynamics of actin regulation were disrupted, leading to abnormal cytoskeletal structures. The presence of these assemblies in brain samples suggests that dysregulation of actin and cofilin could play a key role in the pathogenesis of AD, either as a trigger for further neuronal damage or as a response to the degenerative process. This review explores the current literature and examines apparently contradictory findings regarding the activation and inactivation of the LIMK1-cofilin-actin axis in dendritic spine pathology associated with AD. Specifically, we explored the roles of both activation and inactivation within this pathway, underscoring its complexity and importance in disease progression, suggesting a potential compensatory mechanism, and identifying possible therapeutic targets within this pathway.

## Role of LIMK1 in dendritic spine dynamics

The LIMK family consists of two members: LIMK1 and LIMK2, playing a crucial role in neuronal growth and morphology, synaptic functions, learning and memory [27, 28]. LIMK1 and LIMK2 differ in terms of expression, subcellular localization, and regulatory mechanisms [29]. Due to a palmitoyl motif that ensures its localization and anchoring within the spines, LIMK1 has a unique role in the structural plasticity of dendritic spines, unlike LIMK2 [30]. Indeed, although LIMK2 shares 50% identity overall (70% identity in the kinase domain) with LIMK1 and is present in the brain, it cannot replace LIMK1, as highlighted by the presence of spine abnormalities and cognitive deficits observed in LIMK1 knockout mice and humans with LIMK1 mutations [8, 30–32].

LIMK1 is a ~70 kDa protein that contains a variety of domains, each contributing to its multifaceted role in cellular processes.

The two N-terminal LIM domains, which are zinc finger-like structures, are primarily involved in protein-protein interactions and are known to be critical for the ability of the protein to mediate signal transduction and cytoskeletal organization [33]. Removal of N-terminal LIM domains or mutating conserved cysteines in these domains led to a significant increase in LIMK1 kinase activity, suggesting that the N-terminal LIM domain negatively regulates the kinase activity of LIMK1 by directly interacting with the kinase domain [34].

The two leucine-rich Nuclear Export Signals (NES), short amino acid sequences (236 LDEIDLL; 249 LLQLTL), facilitate the transport of proteins from the nucleus to the cytoplasm. The presence of the Nuclear Localization Signals (NLS) (499 KKPDRKKR) indicates that LIMK1 may shuttle between nuclear and cytoplasm compartments, allowing it to participate in both nuclear and cytoplasmic functions, which could be pivotal for its role in cellular signaling pathways [35]. Indeed, LIMK1 interacts with the cyclic AMP response element-binding protein (CREB) and regulates its activity [28]. This interaction is involved in synaptic plasticity and memory formation. LIMK1 knockout mice show altered long-term memory and synaptic plasticity, which are rescued by enhancing CREB activity [36]. Similarly, basic fibroblast growth factor (bFGF) induces the binding between LIMK1 and CREB, leading to the phosphorylation of CREB, enhancing its ability to stimulate cAMP-responsive element-mediated gene transcription, which is crucial for neuronal differentiation [28].

Additionally, the interaction domains with 14-3-3-ζ protein highlight its regulatory significance, particularly in response to phosphorylation events [37]. The 14-3-3 proteins (seven 14-3-3 identified, i.e., β, γ, ε, η, σ, τ and ζ) are regulatory molecules interacting with phosphorylated serine/threonine residues on their target proteins, influencing various cellular processes such as apoptosis, cell cycle control, and signal transduction [37, 38].

The PDZ domain within LIMK1 aids in anchoring the protein to specific cellular locations, acting as a scaffold for assembling signaling complexes [35].

The serine-threonine kinase domain is pivotal for its enzymatic activity, allowing LIMK1 to phosphorylate substrates like cofilin, thereby regulating actin filament dynamics in spines [8, 30, 32]. Thus, the high specificity of LIMK1 for Ser3 cofilin phosphorylation highlights its critical role in modulating actin dynamics and dendritic spine morphology [11].

Through its diverse domains and interactions with proteins such as 14-3-3, cofilin, Rac and Rho GTPases, ROCK, PAK, α-actinin, and others, LIMK1 orchestrates critical cellular functions, including motility, division, differentiation, and signal transduction.

**Fig. 2:**
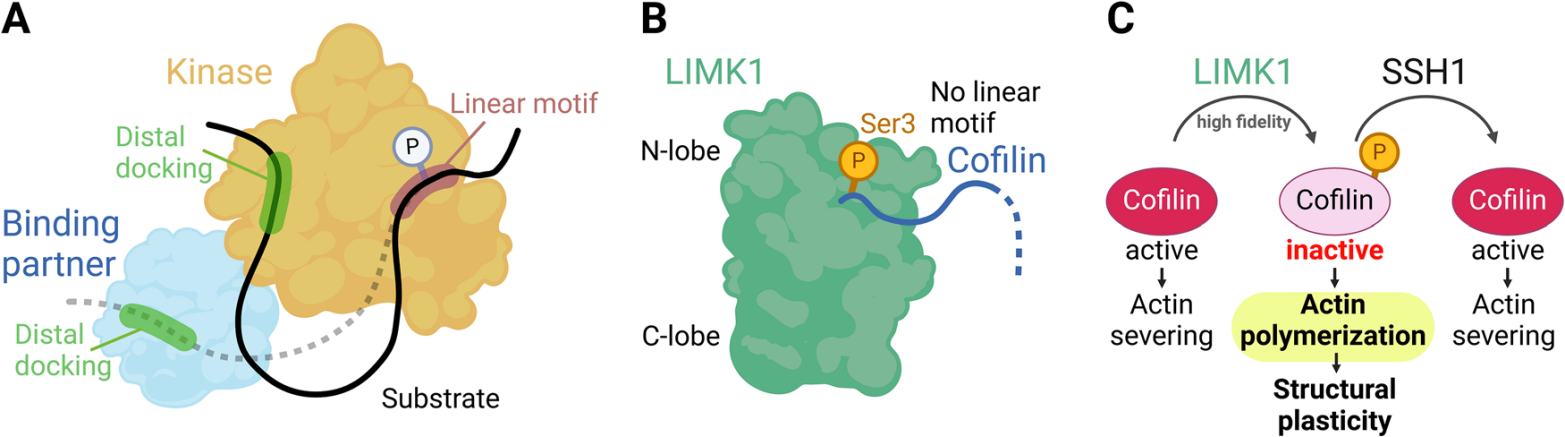
Mechanisms of cofilin regulation and its role in actin dynamics through LIMK1-mediated Ser3 phosphorylation. **A** Cartoon representation of a kinase interacting with a substrate. The kinase engages with the substrate through distal docking and a linear motif, leading to phosphorylation (P) of the substrate. **B** Cartoon of LIMK1 interacting with cofilin. LIMK1 phosphorylates cofilin at Ser3 in the absence of a linear motif, highlighting the peculiarity of its activity. **A**, **B** Modified from Hamil et al. [39]. **C** Schematic diagram of cofilin regulation and its impact on actin dynamics. Active cofilin (unphosphorylated) promotes actin severing. Phosphorylation by LIMK1 inactivates cofilin, leading to actin polymerization (highlighted in yellow) and enhanced structural plasticity. SSH1 dephosphorylates cofilin, restoring its actin-severing activity. Created with BioRender.com.

Of note, the high specificity of the LIMK1-cofilin interaction involves unique features absent in other kinase-substrate pairs [39]. Indeed, the general mechanism of action of the kinase with a substrate involves the presence of two motif interactions: a distal “docking motif” and a phosphosite “linear motif” with the kinase catalytic cleft (Fig. 2) [40]. Distal docking interactions occur outside the kinase catalytic cleft and involve specific binding sites on both the kinase or through regulatory proteins and their substrate [40]. This specificity is achieved through highly selective binding sites that recognize distinct sequences or motifs on the substrate, positioning the substrate in an optimal alignment for phosphorylation, thereby increasing the efficiency of the reaction. The linear motif interactions occur between an extended peptide encompassing residues N- and C-terminal of the phosphoacceptor site and a region adjacent to the kinase catalytic center (Fig. 2).

Hamill and colleagues revealed through co-crystallization studies that LIMK1 interaction with cofilin-1 deviates from these established interaction paradigms. Specifically, LIMK1 does not utilize a typical linear motif nor does it exhibit docking interactions involving additional domains. Instead, LIMK1 directly phosphorylates cofilin at Ser3, located at the N-terminus, without extensive engagement of adjacent residues. The Ser3 residue is precisely positioned within the catalytic site through a complementary interaction between the LIMK1 C-lobe and the cofilin α5 helix, forming a “molecular drill jig” that secures the substrate residue in an optimal orientation for phosphotransfer. This unique mechanism underpins the extraordinary specificity of LIMK1 for cofilin proteins, providing insights into how kinases can achieve high fidelity in substrate selection through specialized structural adaptations [39].

## Role of cofilin in dendritic spine dynamics

Cofilin is a small (~20 kDa) protein identified in the 1980s for its capability to bind actin [41]. The two isoforms of cofilin, cofilin-1 and cofilin-2 are prominent members of the actin-depolymerizing factor (ADF)/cofilin family [42]. These isoforms play distinct roles in cellular processes, with cofilin-1 (non-muscle cofilin) and cofilin-2 (muscle cofilin) exhibiting differential expression and functions [43]. Cofilin-1 plays a pivotal role in the function and plasticity of dendritic spines [44, 45]. Accumulation of cofilin-1 in sub-spinous regions controlling actin filament dynamics, influences spine density, morphology, trafficking of AMPA receptors, and ultimately LTP and its opposite functional phenomenon, long-term depression (LTD), which are key mechanisms underlying learning and memory [42]. LTP and LTD are two key forms of synaptic plasticity, involving the strengthening and weakening of synaptic connections, respectively. Cofilin-mediated regulation of actin dynamics is essential for both LTP and LTD, as the structural changes in dendritic spines depend on the controlled assembly and disassembly of actin filaments [46].

Cofilin binds to actin filaments and promotes their disassembly, a process critical for the dynamic restructuring of the actin cytoskeleton [47]. This actin remodelling is necessary for structural support and enabling changes in spine shape and size, the growth and retraction of dendritic spines, and processes that are fundamental to synaptic plasticity [48].

At low binding densities, cofilin interacts with actin filaments (F-actin) in a way that destabilizes the structure [49]. Cofilin attaches to the sides of the actin filaments and induces a twisting motion. This mechanical stress weakens the bonds between actin subunits, making the filament more susceptible to breaking. As a result, the filament severs into smaller fragments. These fragments can then undergo further disassembly or potentially be reassembled depending on the cellular context and the presence of other regulatory proteins. This severing activity is crucial for the dynamic reorganization of the actin cytoskeleton, which is essential for various cellular processes such as motility, division, and changes in cell shape [50]. Conversely, when cofilin binds to actin filaments at high densities, the scenario changes dramatically. Instead of promoting disassembly, cofilin stabilizes the filaments. In this situation, a high ratio of cofilin molecules to actin subunits leads to the formation of a cofilin-actin complex. This complex stabilizes the filament structure, preventing further severing and disassembly. The stabilization effect arises because the extensive binding of cofilin covers the actin filament protectively, shielding it from the mechanical stresses that would otherwise cause it to break apart [51]. This dual role of cofilin is a fine example of how a single protein can have multiple, context-dependent functions within the cell. At low concentrations, cofilin primary role is to promote the turnover and remodelling of actin filaments, which is vital for quick cellular responses to environmental changes. At high concentrations, cofilin helps to maintain the integrity and stability of actin structures, ensuring that the cytoskeleton remains robust enough to support cellular architecture and function [52]. The ability of cofilin to switch roles based on its binding density adds a layer of regulatory complexity to the control of the actin cytoskeleton, allowing cells to finely tune their structural dynamics according to their specific needs [53].

The ability of cofilin to promote actin assembly or disassembly also depends upon the relative concentrations of other actin-binding proteins [54, 55]. Among these, the Actin-interacting protein 1 (Aip1) can enhance the depolymerizing activity of cofilin, favoring the generation of actin monomers [56]. Moreover, a rise of Aip1 concentration at the dendritic spine has been observed immediately after structural long-term potentiation (sLTP) induction [8], suggesting its synergistic interaction with cofilin in disassembling actin filaments in the early phases of sLTP.

During LTP synapses are strengthened, often resulting in the enlargement of dendritic spines. Cofilin activity must be precisely regulated to allow for the addition of new F-actin, which supports spine growth [47]. In fact, cofilin concentration rapidly increases during the first 20 s after sLTP induction, and, unlike other proteins, its amount remained highly enriched in the spine for up to 30 min. Given that total actin concentration also increases in the spine head during the first seconds/minutes of sLTP [8, 57], cofilin severing activity has been suggested as a key effector facilitating F-actin assembly and spine growth [58–60].

Conversely, during LTD, synapses are weakened, and dendritic spines may shrink or be eliminated. Cofilin facilitates the disassembly of actin filaments, aiding in the retraction of spines and an increase in cofilin-dependent actin severing (dephosphorylation) has been observed to cause spine shrinkage during LTD [61, 62].

**Fig. 3:**
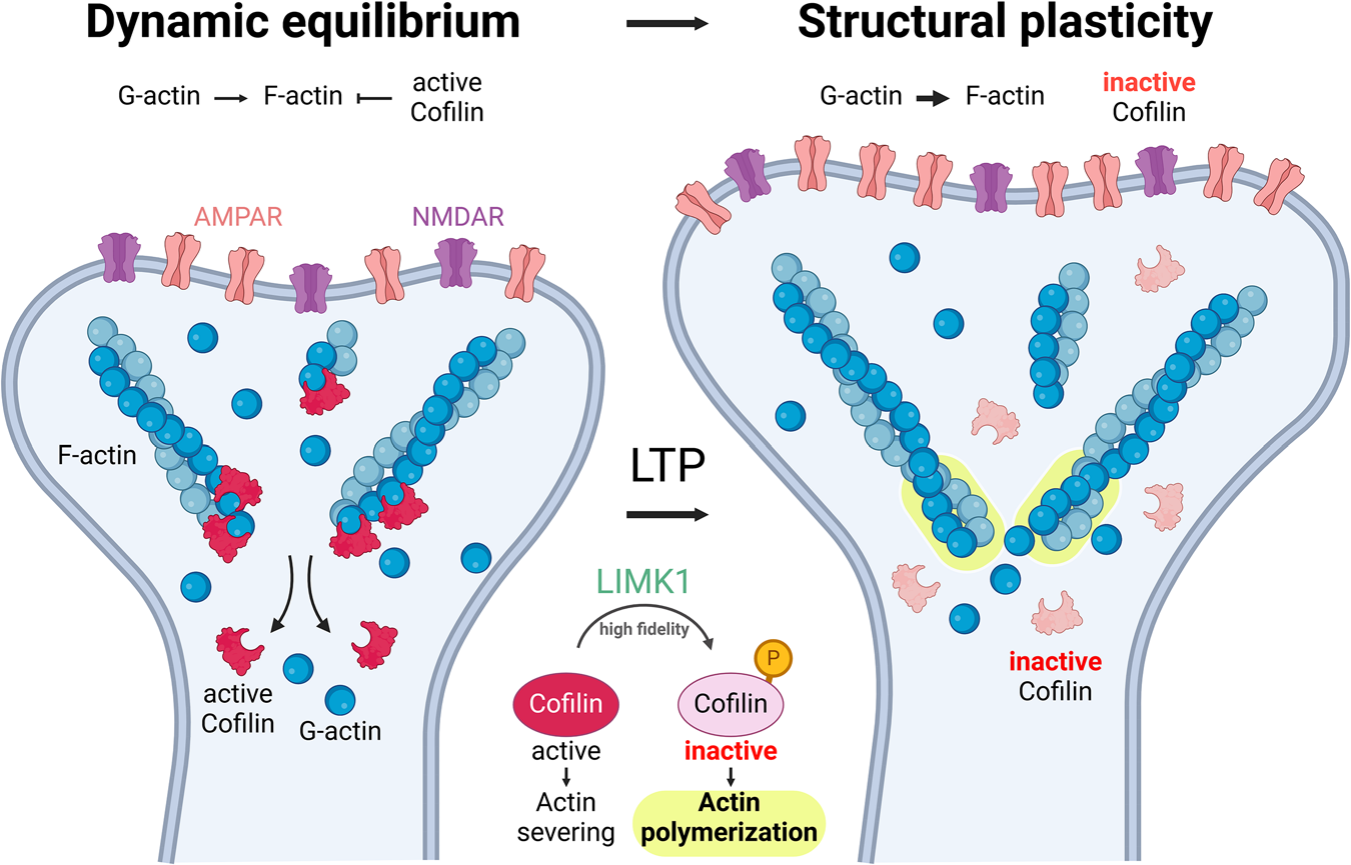
Cofilin-mediated actin polymerization governs the structural plasticity of dendritic spines. Modulation of actin polymerization within dendritic spines through the activity of cofilin. On the left, active cofilin (in red) facilitates the severing and depolymerization of F-actin (blue filaments), releasing G-actin monomers (blue spheres). At rest, a balance between two opposing processes, actin polymerization and cofilin activity, maintains the structure of the spine. AMPAR and NMDAR receptors are illustrated on the synaptic membrane. The right panel shows the transition where LIMK1 phosphorylates cofilin, making it inactive, thereby allowing actin polymerization (highlighted in yellow) to promote the expansion of the dendritic spines, essential for structural changes associated with volume enlargement and the corresponding increase in the number of AMPAR. Created with BioRender.com.

Cofilin activity in dendritic spines is regulated by phosphorylation and dephosphorylation processes. When LIMK1 phosphorylates cofilin at Ser3, it becomes inactive and cannot bind to actin (Fig. 3). This phosphorylation is a critical regulatory mechanism that prevents cofilin from promoting actin filament disassembly. On the other hand, cofilin is reactivated by dephosphorylation through the action of SSH. These phosphatases remove the phosphate group from cofilin, restoring its ability to bind to and sever actin filaments. The balance between LIMK-mediated phosphorylation and SSH-mediated dephosphorylation ensures that cofilin activity is finely tuned in response to cellular needs [63]. This balance between phosphorylated (inactive) and dephosphorylated (active) cofilin is crucial for maintaining normal cellular function. In its inactive, phosphorylated state, cofilin cannot interact with actin filaments, thus preventing unnecessary filament disassembly (Fig. 3). Conversely, when cofilin is dephosphorylated and active, it can promote the turnover of actin filaments, facilitating necessary cellular processes such as synaptic plasticity, cell motility, and structural remodeling [64].

### The role of cofilin in AD pathology

Several evidence support the critical role of cofilin in the pathophysiology of AD, indicating that the cofilin activation/inactivation regulatory mechanism is involved in the cytoskeletal alterations observed in AD [65]. Cofilin accumulation in senile plaques of AD patients and in AD mouse model tissues has been observed [25]. Additionally, the active (dephosphorylated) cofilin can affect axonal trafficking by forming “cofilin rods”, which are aberrant cofilin-actin aggregates contributing to synaptic plasticity deficits [66–68]. Cofilin rods closely colocalized with extracellular Aβ plaques, and subnanomolar concentrations of Aβ dimers/trimers can promote cofilin rod formation [19, 25, 69, 70]. Moreover, it has been suggested that cofilin can coprecipitate with tau and/or other microtubule-associated proteins (MAPs) in cofilin-actin rods [71]. Additionally, both tau and cofilin play a role in mediating mitochondrial and synaptic dysfunction induced by Aβ, and cofilin or tau decreased expression can rescue synaptic plasticity and memory alterations in an APP transgenic mouse model [72, 73].

Besides cofilin rods being constituted by active (dephosphorylated) cofilin, cofilin aberrant phosphorylation has also been implicated in AD pathology. Increased levels of ROCK and corresponding elevated levels of inactive p-cofilin have been observed in AD patient brains and in tissues of APP-expressing mouse models [66, 74, 75]. Collectively, these studies indicate that the regulation of cofilin phosphorylation/dephosphorylation machinery exerts a pivotal role in the synaptotoxic damage observed in neurodegenerative disorders.

## Activation of the LIMK1-cofilin-actin axis in AD

Several studies have highlighted alterations in the activation of the LIMK1-cofilin-actin axis in AD.

Barone et al. [66] corroborate the activation of the LIMK1-cofilin-actin axis in AD, showing increased cofilin phosphorylation and inactivation in both AD patients and mouse models [66]. This process is linked to an age-dependent rise in LIMK1 activity, contributing to synaptic dysfunction. Their study also reveals that inhibiting γ-secretase can prevent cofilin inactivation by activating SSH1, suggesting that γ-secretase not only drives amyloidogenic processes but also modulates cytoskeletal dynamics through the LIMK1-cofilin-actin pathway. In APP/PS1 transgenic mouse brains, p-cofilin levels exhibit a biphasic pattern, with a reduction observed at 4 months of age (corresponding to early pathology) and an increase at 10 months of age (reflecting mid-to-late pathology) [66].

Cofilin function within neurons in AD is complicated by its dual ability to either sever or stabilize actin filaments depending on phosphorylation status [65]. Inactivation of cofilin through LIMK1-mediated phosphorylation leads to actin stabilization, which can protect synapses temporarily, yet excessive stabilization results in pathological structures such as Hirano bodies and cofilin-actin rods. In AD, Hirano bodies and cofilin-actin rods are abundant in regions with severe neurodegeneration, implicating disrupted actin dynamics as a key factor in synaptic impairments and cognitive decline [22, 25].

Interestingly, Schratt et al. [76] identified a miRNA (miR-134), localized in dendritic spines, regulating the expression of LIMK1, thereby controlling dendritic spine size. Indeed, in the absence of synaptic activity, miR-134 can suppress LIMK1 mRNA translation, while synaptic stimulation by BDNF lead to an opposite effect, with enhanced LIMK1 protein synthesis and spine growth [76]. Considering that LIMK1 phosphorylates and activates the CREB/BDNF pathway, a possible role of LIMK1/CREB pathway disregulation in AD can be hypothesised.

Indeed, the role of CREB in cognitive processes is well established: once activated, CREB facilitates the transcription of key proteins necessary for activity-dependent plasticity [77], including BDNF [78], which has been observed to be reduced in AD brains [79, 80]. In addition, CREB has been shown to interact with LIMK1 in cultured cell line [28] and to colocalize with LIMK1 in both hippocampal cultured neurons and brain sections [36]. Notably, it has been shown that LIMK1 deletion in LIMK1 knockout (LIMK1^−/−^) mice is associated with long-term memory deficits, probably due to reduced plasticity-dependent CREB activation [36]. Notably, manipulating CREB, but not cofilin, is sufficient to restore LTP and long-term memory deficits in LIMK1^−/−^ mice, suggesting a mechanism linking LIMK1 and CREB in modulating synaptic plasticity that is independent of the conventional actin regulation process [36]. In the context of neurodegeneration, it could be hypothesised that LIMK1 regulates hippocampal synaptic plasticity and memory functions through two distinct mechanisms mediated by cofilin/actin and CREB, respectively, thus affecting not only spine dynamics but also synaptic plasticity through the modulation of CREB/BDNF pathway.

### The interaction between Aβ and the LIMK1-cofilin axis

**Fig. 4:**
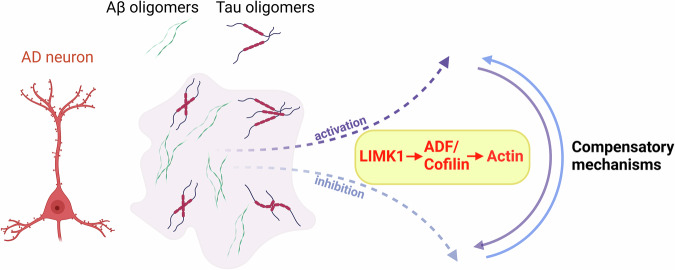
Impaired actin dynamics in Alzheimer’s disease (AD) neurons mediated by amyloid-beta (Aβ) and tau pathology. Cartoon depicting an AD neuron and the impact of Aβ and tau oligomers on LIMK1-cofilin-actin axis in AD spines. Activated LIMK1 phosphorylates ADF/cofilin, leading to the inhibition of its actin-severing activity. Altered actin dynamics result from excessive activation or inhibition of this pathway. The increased cofilin phosphorylation observed in AD may initially act as a compensatory mechanism to restore disrupted actin polymerization. However, persistent dysregulation of the LIMK1-cofilin axis over time can exacerbate synaptic impairment and contribute to further cognitive decline.

Several evidence suggest a link between Aβ and the activation of the LIMK1-cofilin-actin axis within the context of AD. Heredia and colleagues were the first to demonstrate the hyperactivation of the LIMK1 protein, emphasizing its role in Aβ-induced neurodegeneration [17]. The study reveals that the interaction between fibrillar Aβ (fAβ) and murine neuronal cells triggers the canonical phosphorylation of LIMK1, which in turn phosphorylates and inactivates cofilin. This process is implicated in the structural neuronal abnormalities observed in AD pathology, particularly in the formation of dystrophic neurites, pathological features closely associated with cognitive decline in AD (Fig. 4).

The authors provide evidence that this pathway is not only activated in response to Aβ in vitro but also in AD patient brains. Interestingly, the study reports a highly significant increase in the number of neurons positive for phosphorylated LIMK1 in areas of the human brain rich in Aβ accumulation.

In contrast, only few neurons showed a positive staining for phosphorylated LIMK1 in areas free of AD pathology within the same cortex. This highlights a strong association between LIMK1 activation and AD, suggesting the potential role of this pathway in the disease progression. However, it is important to note that the study does not provide data regarding the genetic backgrounds of the selected sporadic AD patients, such as ethnicity, gender, genetic risk scores, APOE genotype, age, AD Braak stage, and Clinical Dementia Rating (CDR) at the time of biopsy. This lack of genetic background information could be a limitation in understanding the broader applicability and significance of the findings. The elevated levels of phosphorylated LIMK1 observed by Heredia et al. suggest a shift towards greater cofilin phosphorylation, thereby increasing the proportion of inactive cofilin in the brain. This shift likely disrupts normal actin dynamics by preventing the necessary turnover of actin filaments. As a result, the structural plasticity of dendritic spines is compromised, probably leading to synaptic dysfunction and contributing to the cognitive deficits characteristic of AD.

Activated cofilin (dephosphorylated) is required for its translocation to mitochondria, where it induces mitochondrial dysfunction, a process associated with apoptosis in AD neurons. Aβ1-42 oligomers are known to increase mitochondrial oxidative stress and cell death, events largely prevented by siRNA knockdown of cofilin or SSH1, illustrating that the oxidative environment in AD accelerates cofilin’s role in neurotoxicity [81, 82]. Upon oxidation, cofilin undergoes disulfide bridging, which reduces its affinity for actin and promotes mitochondrial swelling and cytochrome c release—events that initiate apoptosis [83]. Mitochondrial dysfunction triggered by cofilin activity has downstream effects on cellular energy balance, further implicating cofilin as a contributor to neuronal apoptosis and synaptic dysfunction in AD [19].

Short-term Aβ exposure can increase cofilin phosphorylation, promoting actin stabilization, yet impairing synaptic plasticity [84]. This biphasic pattern suggests that cofilin alternates between states of inactivation and hyperactivation, each stage contributing to neurodegeneration through unique mechanisms. Similarly, Henderson and colleagues [18] demonstrated that Aβ oligomers activated the ROCK pathways, specifically influencing the ROCK2 isoform [18]. This activation leads to the phosphorylation of LIMK1, which in turn phosphorylates and inactivates cofilin. The inactivation of cofilin results in the stabilization of actin filaments, contributing to the degeneration of dendritic spines and the subsequent synaptic dysfunction characteristic of AD [18]. In primary rat hippocampal neurons and in hAPPJ20 transgenic mice, the study showed that increased levels of ROCK2 and phosphorylated LIMK1 correlated with reduced spine density and structural plasticity in neurons. Interestingly, the authors found that treatment with a LIMK1 inhibitor could prevent Aβ42-induced spine loss, highlighting the potential role of LIMK1 as a therapeutic target for protecting synapses from Aβ toxicity. Moreover, live-cell imaging and multielectrode array (MEA) analyses demonstrated that the inhibition of LIMK1 not only preserved dendritic spine density but also protected neurons from Aβ-induced hyperexcitability, which is associated with cognitive decline in AD [18]. This research aligns with the findings from Herskowitz et al. [75] on ROCK2 inhibition, further underscoring the therapeutic potential of targeting specific pathways involved in Aβ-induced synaptic damage. Both studies highlight the critical role of ROCK2 and its downstream effects in the progression of AD, suggesting that selective inhibition of these pathways could offer new strategies for mitigating synaptic and cognitive impairments typical of AD.

Beside ROCK2, also p21-activated kinase 1 (PAK1), an upstream regulator of LIMK1, has been implicated in Aβ signaling and AD pathology. Zhao et al [85] demonstrated that PAK signaling in markedly reduced in post-mortedm brains of AD patients, together with altered cofilin and debrin expression, suggesting that the defects in this actin-regulatory machinery could be has a key role in dendritic and synaptic dysfunction in AD. Additionally, the same authors, using both in vitro and in vivo models, demonstrates that the abnormalities of PAK1/cofilin/debrin pathway are causally related with Aβ pathology. Indeed, the authors showed that Aβ oligomers can be responsible for PAK defects, leading to cofilin and drebrin alterations and, further, that the expression of active PAK can exert a protective effect against the Aβ oligomers [85].

The interplay between LIMK1 activation, cofilin phosphorylation, and actin stabilization or severing reflects an adaptive, yet ultimately maladaptive, response to AD-specific stressors [86]. Studies suggest that LIMK1 activation in AD might initially serve a compensatory function aimed at mitigating cofilin activity, a process that limits cofilin’s potential to destabilize actin structures [16]. Increased LIMK1 activity leads to the phosphorylation and inactivation of cofilin, promoting actin stabilization, a vital response for maintaining synaptic integrity in neurons subjected to Aβ-induced stress. This inactivation is particularly relevant in early AD, where the brain response to amyloid stress may favor mechanisms that stabilize dendritic spines, thereby preserving essential synaptic structures [87].

In cellular studies, β1-integrin, a receptor crucial for synaptic maintenance, has been shown to mediate Aβ-induced cofilin dephosphorylation through the activation of SSH. This dephosphorylation leads to cofilin activation and actin severing, yet the observed compensatory increase in LIMK1 activity in AD brains appears to counteract this activation, suggesting that the pathway regulation is context-dependent [19]. Aβ1-40 and Aβ25-35 fibrils specifically activate LIMK, leading to cofilin inactivation in dystrophic neurites, a mechanism that may serve to prevent excessive actin severing during initial stages of Aβ pathology [17]. This points to a biphasic, region-specific activation pattern, where in early AD, the LIMK1-cofilin axis potentially stabilizes actin to limit initial synaptic disruption and maintain dendritic spine integrity.

In AD, however, cofilin-actin rod formation, often triggered by Aβ dimers and trimers, is markedly increased. These rod-like structures accumulate in dendritic spines, impairing synaptic function and contributing to memory deficits [67]. The fourfold increase in cofilin rods observed in AD correlates with tauopathy severity, indicating that the LIMK1-cofilin pathway may link Aβ and tau pathologies to synaptic dysfunction in AD [88].

The role of LIMK1 and cofilin in AD is complicated by context-dependent regulatory mechanisms, with observed differences in pathway activity likely influenced by factors such as disease stage, neuronal type, and local concentrations of neurotoxic proteins. For example, areas of the brain with high Aβ concentrations might experience heightened cofilin activation due to SSH-mediated dephosphorylation, leading to localized actin destabilization and synaptic damage [70, 81]. Conversely, other regions may exhibit increased LIMK1 activity, which promotes cofilin inactivation and actin stabilization, potentially forming protective but ultimately pathological cofilin-actin aggregates [21].

Further complicating the role of the LIMK1-cofilin-actin axis is evidence suggesting a biphasic regulation pattern throughout AD progression. In early disease stages, increased LIMK1 phosphorylation of cofilin may represent an adaptive response to Aβ-induced stress, stabilizing actin filaments to preserve synaptic function [17]. However, as the disease burden increases, this pathway may shift towards cofilin hyperactivation, leading to actin severing and the loss of synaptic plasticity [82].

### The interplay between cofilin and Tau

The link between cofilin and tau, two proteins that both regulate microtubule and actin cytoskeleton dynamics, is central to understanding AD pathology. While cofilin destabilizes actin filaments, tau stabilizes microtubules, yet in AD, both proteins contribute to neurodegeneration through the formation of pathological aggregates [15]. In mouse models, tau and cofilin interactions appear to displace tau from microtubules, inhibiting tau-induced microtubule assembly and axonal transport, mechanisms critical for neuronal communication and synaptic integrity [89]. The study by Woo et al. [15] offers significant insights into how tau mutations can lead to cofilin dysregulation. Using the P301S tauopathy mouse model, the researchers demonstrated that tau mutations associated with tauopathies, such as AD, result in aberrant regulation of cofilin. In the P301S mouse model, the presence of mutant tau led to increased levels of phosphorylated cofilin, indicating an imbalance towards the inactive form of cofilin. This was accompanied by a reduction in actin filament turnover, which is crucial for maintaining the dynamic restructuring required for proper synaptic function [15].

In another recent study, the authors investigated how activated cofilin influences tau pathology through its effects on microtubule dynamics. One of the key findings is that activated cofilin competes with tau for binding to microtubules [15]. Normally, tau stabilizes microtubules, which are essential for maintaining neuronal structure and function. However, when cofilin is activated, it binds to microtubules and displaces tau, leading to destabilization of the microtubule network. This displacement impairs tau capability to promote microtubule assembly, thereby contributing to the degeneration of neurons observed in AD. The study also demonstrates that reducing cofilin levels in a mouse model of tauopathy (Tau-P301S mice) can mitigate the harmful effects of tau on microtubules. In these mice, genetic reduction of cofilin restored normal microtubule dynamics, reduced tau pathology, and improved neuronal function [15]. This was evidenced by the recovery of neurite outgrowth and axonal transport in neurons, as well as the reduction of synaptic deficits and movement impairments in transgenic *C. elegans* expressing human tau. It was found that only the active form of cofilin, not the inactive form, binds to microtubules and disrupts their stability. This finding suggests that the activation state of cofilin is crucial for its role in promoting tauopathy, and targeting this activation could be a potential therapeutic strategy for AD and related tauopathies [15].

## Inhibition of the LIMK1-cofilin-actin axis in AD

Targeting cofilin regulation through LIMK1 inhibitors or SSH activators presents a promising therapeutic strategy for AD; however, significant challenges remain in translating these findings into clinical approaches, particularly because other scientific evidence suggests the opposite, that is the excessive inactivation of the LIMK1-cofilin-actin axis may be responsible for the adverse effects observed in AD neurons. This dual perspective underscores the complexity of the pathway and the need for a nuanced approach when considering therapeutic interventions.

In 2007, Shankar and colleagues focused on how different forms of Aβ, i.e., monomeric and oligomeric (dimeric and trimeric) forms of the protein, induced a progressive loss of synapses [20]. In rat hippocampal slices, the authors observed that exposure to oligomeric Aβ led to a marked decrease in the density of dendritic spines [20]. This spine loss was accompanied by a reduction in the number of electrophysiologically active synapses, suggesting that Aβ oligomers directly compromise synaptic integrity [20]. Interestingly, the study also found that this synapse loss was reversible in the presence of Aβ-specific antibodies or small-molecule inhibitors of Aβ aggregation [20]. Aβ oligomers exerted their deleterious effects through NMDARs, reducing Ca^2+^ influx, a critical signal for synaptic strength and stability. The involvement of other molecules, such as calcineurin and cofilin, further pointed to a specific signaling pathway through which Aβ oligomers mediate their effects, aligning with pathways known to be involved in synaptic depression and LTD, a process that leads to the weakening and removal of synaptic connections [20].

Of note, the study found that expression of the S3D phosphomimetic mutant of cofilin, which renders it constitutively inactive, prevented the loss of dendritic spines typically induced by Aβ oligomers [20]. By rendering cofilin inactive, the authors effectively blocked the cascade that leads to actin depolymerization and subsequent spine shrinkage and loss, which are characteristic of LTD.

A recent study [16], investigated the effects of overexpressing LIMK1 in the hippocampal excitatory neurons of APP/PS1 mice, a commonly used model for AD, which exhibits significant reduced levels of phosphorylated cofilin [16], potentially leading to destabilization of the actin cytoskeleton and contributing to synaptic degradation. The overexpression of LIMK1, obtained using AAV vectors, increases significantly cofilin phosphorylation. By increasing LIMK1 levels, the study effectively inactivated cofilin through phosphorylation, thereby stabilizing the actin cytoskeleton and preserving synaptic structures. Moreover, electrophysiological studies showed that APP/PS1 mice, which normally exhibit impaired synaptic plasticity as evidenced by reduced LTP, experienced a restoration of LTP to near-normal levels following LIMK1 overexpression [16]. This suggests that the dysregulation of the cofilin pathway, which contributes to synaptic dysfunction in AD, can be corrected by enhancing LIMK1 activity. Indeed, behavioral tests further demonstrated that the overexpression of LIMK1 in the hippocampus of APP/PS1 mice resulted in improved performance in memory tasks, which are typically impaired in AD models. Interestingly, while the overexpression of LIMK1 in wild-type mice did not adversely affect synaptic plasticity, it did lead to some unexpected effects on social behavior, such as reduced performance in social memory tasks. This finding suggests that while LIMK1 modulation can be beneficial in the context of AD, it must be carefully balanced, as excessive LIMK1 activity might have unintended consequences on social behaviors. This highlights that the development of new strategies able to obtain a controlled spatiotemporal protein expression (i.e., by using a chemogenetic approach) is crucial from a translational point of view [90]. Notably, a downregulation of LIMK1-cofilin activity has been recently observed in human neurons derived from reprogramming skin biopsy from sporadic AD patients [91].

Woo et al. investigated the role of RanBP9, a scaffolding protein, in the pathogenesis of AD, particularly focusing on its interaction with cofilin [70]. RanBP9 is crucial in mediating the neurotoxic effects of Aβ, indeed, its overexpression increased Aβ production in both cell lines and transgenic mouse models, exacerbating AD pathology [70]. RanBP9 was found to promote the activation of cofilin through the regulation of SSH1. Once dephosphorylated and activated, cofilin translocates into the mitochondria, leading to mitochondrial dysfunction and to the formation of cofilin-actin rods, which are associated with synaptic damage and neurodegeneration [70]. Thus, reducing RanBP9 levels, either through genetic manipulation or RNA interference, significantly decreased cofilin activation, prevented its translocation to mitochondria, and minimized the formation of cofilin-actin rods in neurons. These changes were associated with a reduction in synaptic loss and a partial rescue of synaptic plasticity, as evidenced by improvements in LTP in brain slices from APP/PS1 transgenic mice with reduced RanBP9 levels. Behavioral assessments revealed that reducing RanBP9 levels in APP/PS1 mice partially rescued deficits in contextual memory, a hippocampus-dependent cognitive function [70]. By modulating the RanBP9-SSH1-cofilin pathway, it may be possible to alleviate the synaptic and cognitive deficits associated with the disease. The study also showed that RanBP9 reduction protected against neuroinflammation and decreased the accumulation of Aβ plaques in the brains of APP/PS1 mice. This was accompanied by a reduction in the loss of postsynaptic proteins, such as PSD95 and Drebrin, which are critical for maintaining synaptic integrity and function. Findings suggest that targeting RanBP9 could be a promising therapeutic strategy for treating AD, particularly in its early stages. The study highlights the complex interplay between actin dynamics, mitochondrial function, and synaptic health in the progression of AD and underscores the potential of RanBP9 as a critical target for intervention.

Similarly, very recently, Cazzaro et al. [81] demonstrated how SSH1 affected the neuroprotective functions of Nuclear factor erythroid 2-related factor 2 (Nrf2), a redox-sensitive transcription factor playing a pivotal role in oxidative stress regulation [81]. Indeed, Nrf2 responds to oxidative stress by translocating into the nucleus, where it induces the expression of an array of antioxidant response element (ARE)–dependent genes. Authors demonstrated that in experimental models of AD, SSH1 becomes abnormally activated and binds to Nrf2, preventing it from entering the nucleus and performing its protective functions.

## Conclusions

In this review, we summarized current findings on LIMK1-cofilin-actin signaling in regulating spine dynamics, focusing on its role in AD pathology and emphasizing the dual role of cofilin in stabilizing and severing actin filaments. The LIMK1-cofilin-actin pathway presents a complex and dynamic role in the pathogenesis of AD, where it appears to mediate both neuroprotective and neurodegenerative mechanisms across different stages of disease progression. Interestingly, cofilin inactivation, marked by increased phosphorylation, may also contribute to AD pathology in a biphasic manner. This biphasic pattern suggests that cofilin alternates between states of inactivation and hyperactivation, each stage contributing to neurodegeneration through unique mechanisms. This regulatory axis, critical for actin cytoskeleton stability and synaptic plasticity, can function in opposite ways depending on environmental factors within the brain, disease stage, neuronal type, and regional variations in Aβ and tau accumulation (Fig. 4). Modulating this pathway, particularly by inhibiting cofilin activation, may offer a means to protect against the synaptic and cognitive impairments characteristic of AD.
